# Dry Eye Disease following Refractive Surgery: A 12-Month Follow-Up of SMILE versus FS-LASIK in High Myopia

**DOI:** 10.1155/2015/132417

**Published:** 2015-11-15

**Authors:** Bingjie Wang, Rajeev K. Naidu, Renyuan Chu, Jinhui Dai, Xiaomei Qu, Hao Zhou

**Affiliations:** ^1^Key Myopia Laboratory of Chinese Health Ministry, Department of Ophthalmology, Eye & ENT Hospital, Fudan University, No. 83, Fenyang Road, Shanghai 200031, China; ^2^The University of Sydney, Camperdown, NSW 2006, Australia

## Abstract

*Purpose.* To compare dry eye disease following SMILE versus FS-LASIK.* Design.* Prospective, nonrandomised, observational study.* Patients.* 90 patients undergoing refractive surgery for myopia were included. 47 eyes underwent SMILE and 43 eyes underwent FS-LASIK.* Methods.* Evaluation of dry eye disease was conducted preoperatively and at 1, 3, 6, and 12 months postoperatively, using the Salisbury Eye Evaluation Questionnaire (SEEQ) and TBUT.* Results.* TBUT reduced following SMILE at 1 and 3 months (*p* < 0.001) and at 1, 3, and 6 months following FS-LASIK (*p* < 0.001). TBUT was greater following SMILE than FS-LASIK at 3, 6, and 12 months (*p* < 0.001, *p* < 0.001, and *p* = 0.009, resp.). SEEQ scores increased (greater symptoms) following SMILE at 1 month (*p* < 0.001) and 3 months (*p* = 0.003) and at 1, 3, and 6 months following FS-LASIK (*p* < 0.001). SMILE produced lower SEEQ scores (fewer symptoms) than FS-LASIK at 1, 3, and 6 months (*p* < 0.001).* Conclusion.* SMILE produces less dry eye disease than FS-LASIK at 6 months postoperatively but demonstrates similar degrees of dry eye disease at 12 months.

## 1. Introduction

Dry eye disease is a common ocular surface disease and plays a significant role in the ocular comfort and visual performance of patients, with the potential to have a great impact on their quality of life [1–6]. Dry eye is known to be a frequently reported and observed finding following refractive surgery, particularly in the period immediately following surgery [7–12]. With refractive surgery cases increasing in number, dry eye is becoming an increasing challenge for refractive surgeons to overcome, with a large proportion of patients experiencing dry eye symptoms to varying degrees [3, 10, 13–18]. Dry eye has also been associated with a delayed wound healing response and may predispose patients to refractive regression in moderate to severe cases [7, 15].

While the pathophysiology of this complication is still evolving, a number of theories have been proposed to explain why dry eye occurs following refractive surgery, including exacerbation of preexisting dry eye disease [12], medicamentosa from postoperative medications [19, 20], and damage to conjunctival goblet cells increasing tear hyperosmolarity and inflammation [19, 21–23]. The interaction between the ocular surface and eyelids is an important factor in maintaining tear production and flow, which is also altered following surgery [10, 24]. Perhaps the biggest factor, however, is the impact surgery has on corneal nerves and sensation [19, 21, 25, 26]. Intact corneal sensation is required for adequate blink frequency and tear production, and corneal denervation resulting from disruption and damage to corneal nerves has been shown to play a significant role in the development of dry eye disease following refractive surgery [27–29].

Laser-assisted in situ keratomileusis (LASIK) continues to be a popular refractive surgical option [18, 30]; however, almost half of all LASIK patients continue to report dry eye symptoms following surgery [8]. The introduction of the femtosecond laser (FS) has seen FS-LASIK become a more accurate and safe surgical option, with a reduced rate of dry eye disease, which is likely due to reduced neurotrophic effects on the corneal nerves during formation of the corneal flap [22]. A recent advancement in refractive surgery has been small-incision lenticule extraction (SMILE), which was established as a “flapless” procedure in which an intrastromal lenticule is cut by a femtosecond laser and manually extracted through a peripheral corneal tunnel incision. The refractive predictability, safety, and patient satisfaction of SMILE are comparable to FS-LASIK. SMILE has the benefit of being minimally invasive, with a lesser degree of damage to the cornea and corneal nerves, and may therefore result in fewer complications and reduced symptoms of dry eye [9]. The key difference between FS-LASIK and SMILE and their impact on corneal innervation may lie in the fact that FS-LASIK affects the epithelium and anterior stroma, thus resulting in greater resection of the sensory nerves of the cornea [19–21], while SMILE affects the posterior stromal bed with relatively greater preservation of the corneal subbasal nerve plexus [9].

Few studies exist in the literature investigating the long-term effects of refractive surgery, specifically comparing both SMILE and FS-LASIK, on the development of dry eye syndromes. In this prospective observational study, we present the findings of the objectively measured clinical signs and subjective reporting of dry eye symptoms following SMILE versus FS-LASIK for the correction of myopia in a large group of demographically similar patients over a period of 12 months postoperatively.

## 2. Methods

### 2.1. Setting and Design

This institutional, prospective, observational study was approved prospectively by the institutional review board of The Eye and ENT Hospital of Fudan University. Written informed consent was obtained from all patients prior to participating in the study. The study adhered to the guidelines and principles of the Declaration of Helsinki.

### 2.2. Patients

Patients who attended The Eye and ENT Hospital of Fudan University, Shanghai, China, between the period of January 2012 and January 2014, for refractive treatment of their myopia were recruited.

Inclusion criteria included patient aged over 18 years, Spherical Equivalent (SE) refractive error ≥ −6.00 D, a stable refractive error in the last 2 years, no contraindications to laser refractive surgery, and no previous history of dry eye disease. Additionally, prior to surgery, patients completed a dry eye questionnaire (The Salisbury Eye Evaluation) and only those who yielded a total score of 0 were included. Patients were excluded if they had undergone any ocular surgery in the past 6 months or were using medication that could interfere with the ocular surface. A complete dilated ophthalmic examination was performed to assess the patient's suitability for either SMILE or FS-LASIK. Central corneal thickness (CCT) was determined with a Pentacam system (Typ70700; Oculus; Wetzlar, Germany). After the nature of the two procedures was explained, the patients chose the type of surgery they wished to undergo.

In total, 90 patients who completed 12 months of follow-up were included in this study. 47 patients underwent SMILE procedures (SMILE group) while 43 patients underwent FS-LASIK procedures (FS-LASIK group). The mean age of patients undergoing SMILE was 25.21 ± 6.51 years old, which was not significantly different to the mean age of patients undergoing FS-LASIK, which was 24.72 ± 6.53 years old (*p* = 0.722). The mean preoperative SE was −7.46 ± 1.11 D in the SMILE group and −7.44 ± 1.13 D in the FS-LASIK group, with no significant difference between the two groups. The mean preoperative TBUT was 9.87 ± 1.57 seconds in the SMILE group and 9.56 ± 1.35 seconds in the FS-LASIK group, again with no significant difference between the two groups (*p* = 0.948). Written informed consent was obtained from each patient after the details of the study were fully explained.

#### 2.2.1. Tear-Film Breakup Time (TBUT)

TBUT was assessed prior to surgery and was repeated at 1 month, 3 months, 6 months, and 12 months after surgery. TBUT was assessed with fluorescein paper strips that were wetted with unpreserved saline solution. One drop was instilled in each eye in the lower conjunctival sac, and the patient was instructed to blink several times. A cobalt filter was attached to a slit-lamp biomicroscope, and the time it took from a complete blink until the first signs of a break in the tear film was recorded. The test was repeated 3 times and averaged. The same observer performed the test.

#### 2.2.2. The Salisbury Eye Evaluation Questionnaire for Dry Eye Symptoms

The Salisbury Eye Evaluation Questionnaire, translated into Chinese, was given to each subject for self-evaluation of dry eye symptoms before operation and at 1, 3, 6, and 12 months after operation. The questionnaire contains 6 items pertaining to dry eye symptoms. Questions include the following: (1) Do your eyes ever feel dry? (2) Do you ever feel a gritty or sandy sensation in your eye? (3) Are your eyes ever red? (4) Do your eyes ever have a burning sensation? (5) Do you notice much crusting on your lashes? (6) Do your eyes ever get stuck shut in the morning? The subject answers each question on the questionnaire based on how often they experience these symptoms as rarely, sometimes, often, or all the time. Symptoms that were experienced often or all the time were given a score of 1, and the other two responses were given a score of 0. The scores were added up to give a total score for each subject.

### 2.3. Surgical Technique

All surgeries were performed under local anesthesia by one surgeon (Hao Zhou) with patients undergoing either SMILE or FS-LASIK.

SMILE was performed using the VisuMax femtosecond laser system (Carl Zeiss Meditec) with a repetition rate of 500 kHz, pulse energy of 185–190 nJ, intended cap thickness of 100–120 *μ*m, cap diameter of 7.5 mm, lenticule diameter of 6.1 to 6.6 mm (depending on the refractive error), and a 90°-angle side cut with a circumferential length of 2.1 mm at the superior position.

FS-LASIK was performed with the VisuMax system for flap creation followed by Mel 80 excimer laser (Carl Zeiss Meditec) for stromal ablation, with an intended flap thickness of 95 *μ*m and pulse energy of 185 nJ. The hinge was located at the superior position.

A standard postoperative topical steroid (Fluorometholone 0.1%) was tapered over 30 days; topical antibiotic (Tobramycin 0.003%) QID for 7 days, and unpreserved ocular lubricant 4 times a day was prescribed for a month.

### 2.4. Statistical Analysis

In all cases, only data from the first eye (right eye) on which the procedure was performed was used in the statistical analysis. The sample size of this study was determined based on the standard deviation reported from a previous study [9], with the significance level set at *α* = 0.05 (two tailed) and a power of 90%, and a sample size of at least 38 was required in each group. Allowing for a 10% dropout rate, at least 84 subjects were required. All statistical analyses were performed with a statistics program (SPSS 19.0 IBM Corporation, Armonk, NY, USA). Independent-samples *t*-test was used to compare the differences between groups. One-way repeated measures ANOVA test was used to compare TBUT change and SEEQ score change within groups over time. Tukey's honestly significant difference (HSD) post hoc test was performed to evaluate the differences in parameters between groups. Spearman's correlation test was used to assess relationship between TBUT and SEEQ scores. *p* < 0.05 was considered significant.

## 3. Results

In total, 90 patients were recruited for the study, with a total of 90 eyes (the first eye to have surgery performed for each patient) included in the analysis. There were a total of 47 eyes in the SMILE group and 43 eyes in the FS-LASIK group. There were no significant differences between the two groups preoperatively in terms of age, SE refractive error, central corneal thickness (CCT), or preoperative TBUT. Demographic data for all subjects included in this study is outlined in Table 1.

Objective surgical changes in corneal parameters were similar between the two groups, with no significant difference in lenticule thickness/ablation depth between the two groups (Table 2).

### 3.1. Tear-Film Breakup Time (TBUT)

Preoperatively, there was no significant difference in TBUT between the SMILE and FS-LASIK groups (9.87 ± 1.57 seconds and 9.56 ± 1.35 seconds, resp., *p* = 0.313). One-way ANOVA showed that there was a statistically significant difference in TBUT between preoperative values and the different follow-up time periods, for both SMILE (*F*(4,230) = 79.673, *p* < 0.001) and FS-LASIK (*F*(4,210) = 55.531, *p* < 0.001).

**Figure 1:**
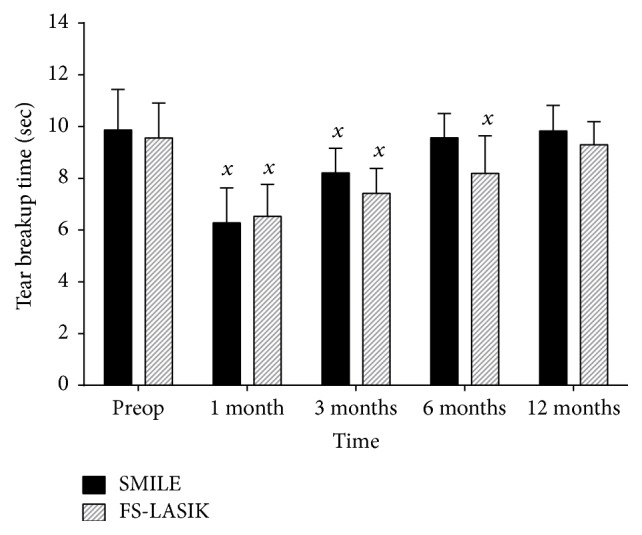
Tear-film breakup time (TBUT) in SMILE and FS-LASIK groups before operation, 1, 3, 6, and 12 months after operation. “*x*”: statistically significantly less than preoperative values, *p* < 0.05.

Post hoc tests showed that, at 1 and 3 months after operation, there was a statistically significant decrease in TBUT from preoperative values in the SMILE group (6.28 ± 1.35, *p* < 0.001, and 8.21 ± 0.95, *p* < 0.001, resp.), before returning to preoperative values by 6 and 12 months (9.57 ± 0.93, *p* = 0.740, and 9.83 ± 0.99, *p* = 1.00, resp.). In the FS-LASIK group, TBUT was statistically significantly reduced from preoperative values at 1 month, 3 months, and 6 months postoperatively (6.53 ± 1.24, *p* < 0.001, 7.41 ± 0.96, *p* < 0.001, and 8.18 ± 1.45, *p* < 0.001, resp.), before returning to preoperative values at 12 months (9.30 ± 0.89, *p* = 0.826) (Figure 1).

Between the two procedures, TBUT was not statistically significantly different at 1 month postoperatively (*p* = 0.348); however, at 3, 6, and 12 months postoperatively, TBUT was statistically significantly greater in the SMILE group than the FS-LASIK group (*p* < 0.001, *p* < 0.001, and *p* = 0.009, resp.) (Table 3).

### 3.2. Salisbury Eye Evaluation Questionnaire

The Salisbury Eye Evaluation Questionnaire (SEEQ) was used to assess a patient's subjective reporting of dry eye symptoms, with a higher score indicating a greater degree of experienced dry eye symptoms. Preoperative scores were 0, per the inclusion criteria. One-way ANOVA testing found a statistically significant difference in SEEQ scores within groups over the time period of review for both SMILE (*F*(4,230) = 23.127, *p* < 0.001) and FS-LASIK (*F*(4,210) = 91.161, *p* < 0.001).

**Figure 2:**
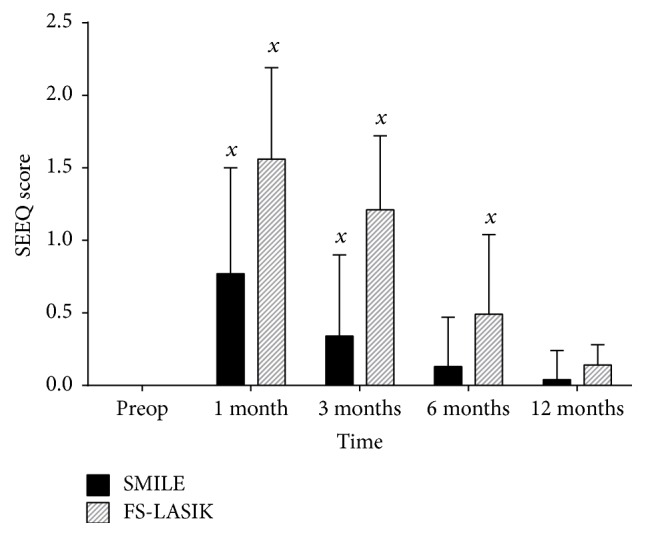
Salisbury Eye Evaluation Questionnaire results in SMILE and FS-LASIK groups at 1, 3, 6, and 12 months after operation. “*x*”: statistically significantly greater than preoperative values, *p* < 0.05.

Post hoc tests showed that, in the SMILE group, SEEQ scores were statistically significantly higher at 1 month (*p* < 0.001) and 3 months (*p* = 0.003) after operation than preoperative values. By 6 and 12 months, this difference was no longer statistically significant (*p* = 0.640 and *p* = 0.991, resp.). For FS-LASIK, post hoc test evaluation found that SEEQ scores at 1 month, 3 months, and 6 months after operation were statistically significantly higher than preoperative values (*p* < 0.001 for all 3 follow-up time periods). By 12 months, this difference was no longer found (*p* = 0.636) (Figure 2).

Postoperatively at the 1-, 3-, and 6-month follow-up intervals, the SEEQ score was higher in the FS-LASIK group than the SMILE group (*p* < 0.001 for all 3 follow-up time intervals). 12 months after operation, this difference was no longer statistically significant (*p* = 0.109) (Table 4).

Spearman's correlation test revealed a moderate negative correlation between SEEQ scores and TBUT at 1 month after operation for the SMILE group (*r*_*s*_ = −0.599, *p* < 0.001) as well as in the FS-LASIK group (*r*_*s*_ = −0.518, *p* < 0.001).

## 4. Discussion

Dry eye disease continues to be a common complication of refractive surgery, affecting not only the ocular comfort of patients, but also their visual quality [8, 31]. This can have a direct impact on their overall satisfaction and quality of life following surgery. Although a frequently noted condition, dry eye disease remains a complex syndrome with a wide-ranging spectrum of clinical signs and subjective symptoms that do not always show a great degree of correlation [4, 32]. While there exist several clinical measures to diagnose and monitor the severity of dry eye disease, it is difficult to fully understand the impact it has on a patient, as many patients that show early clinical signs of dry eye disease may be asymptomatic, while others may report symptoms greater than their clinical signs may suggest, or without any tissue damage at all [32]. Assessment of dry eye disease should, therefore, consist of both clinical examination and subjective self-reporting of symptoms by patients, ideally through the use of a dry eye questionnaire. The purpose of this study was to investigate and compare the long-term dry eye outcomes up to 12 months following SMILE and FS-LASIK for the correction of high myopia, using both clinical (TBUT) and subjective (SEEQ) measures of dry eye disease.

Dry eye symptoms are often considered a transient occurrence, occurring in the vast majority of patients in the short-term following refractive surgery [9, 15, 17]. Many studies have shown an increase in dry eye symptoms in the period immediately following refractive surgery, which often improves within three to nine months postoperatively [7, 9–11, 13–17, 19, 21, 22, 30, 31, 33–36]. In the present study, we investigated the objectively measured clinical signs and subjective reporting of dry eye symptoms following SMILE and FS-LASIK for the correction of high myopia in a large group of demographically similar patients over a longer period of 12 months postoperatively. The main outcome measures of interest were the TBUT as a clinical marker for dry eye disease and the Salisbury Eye Evaluation Questionnaire as a subjective indicator of a patient's experience of dry eye symptoms, with a comparison of both measures between two highly affective refractive procedures, SMILE and FS-LASIK. The use of both of these measures provides a good representation of dry eye disease. TBUT has been shown to be both sensitive and accurate as a noninvasive method of dry eye diagnosis [37], and dry eye questionnaires have been shown to represent the true degree of morbidity of the disease as experienced by patients [2, 4, 32, 37].

The present study demonstrated that both the SMILE and FS-LASIK procedures resulted in changes in both the clinical and subjective markers of dry eye, with a transient increase in dry eye disease in both groups. A reduction in TBUT was observed for both the SMILE and FS-LASIK groups following surgery at 1 and 3 months postoperatively. However, this change was only transient, as the TBUT had recovered to preoperative levels for patients that underwent SMILE by 6 months postoperatively, whereas for patients that underwent FS-LASIK, this recovery did not occur until 12 months postoperatively.

These results suggest that SMILE may be superior to FS-LASIK, inducing a shorter duration of tear-film disturbance and leading to a quicker recovery of tear-film function postoperatively. Our results also indicate that this advantage of the SMILE procedure was noted subjectively with the patient's experience of dry eye symptoms, as demonstrated by the results of the SEEQ. Patients in the SMILE group reported lower SEEQ scores (fewer dry eye symptoms) compared to patients in the FS-LASIK group at 1, 3, and 6 months postoperatively, before equalizing at 12 months postoperatively. Therefore, patients who underwent SMILE were less prone to dry eye symptoms, as assessed with both clinical and subjective measures, than those who underwent FS-LASIK in the first 6 months following surgery, but they demonstrated similar degrees of dry eye disease after 12 months of follow-up.

Several hypotheses have been proposed to explain the pathophysiology underlying the development of dry eye disease following refractive surgery. Changes in corneal innervation and sensitivity induced by refractive surgery are key in understanding the pathogenesis of dry eye disease and revolve around the idea that corneal sensitivity is reduced due to transection of the corneal nerves, thus resulting in dysfunction of the cornea-lacrimal gland functional unit [19, 21]. Transection of the sensory nerves of the cornea, as it occurs during FS-LASIK, is thought to lead to a decrease in the innervation to the autonomic nerve fibres supplying the lacrimal gland that would otherwise stimulate tear production via the neural reflex arc [19, 21]. This change may result in tear-film dysfunction via a number of mechanisms, including changes in the composition of the tears, ocular surface changes, and decreased blink frequency [21, 23].

There has been increasing evidence supporting the theory that damage to the corneal nerve density occurs following refractive surgery, particularly affecting the subbasal nerve plexus [19, 21, 24, 27, 38–40]. The main consequence of this change in nerve density is a reduction in corneal sensitivity. This results in a hypoesthetic cornea and is likely the key factor in the development of postrefractive dry eye disease [13, 23, 25, 26, 35, 41, 42]. With the aid of in vivo confocal microscopy, Denoyer et al. demonstrated that SMILE preserved the corneal subbasal nerve plexus better than LASIK [9]. They found a greater nerve density, number of long nerve fibres and nerve fibre branchings in patients that underwent SMILE compared to those that underwent LASIK. They also found that corneal sensitivity was greater in the SMILE group in the short-term but found no significant difference between SMILE and LASIK after 6 months after surgery [9]. This loss in nerve fibre density does start to regenerate months after surgery, with almost complete recovery by 2 to 5 years postoperatively [21, 43–45].

The degree of injury to corneal nerves is understandably expected to be different between the two surgical procedures, owing to the differing nature of each procedure. The two procedures differ in the method of ablation and the layers of the cornea affected, with FS-LASIK affecting the epithelium and anterior stroma, with the creation of a flap, while SMILE mainly affects the posterior stromal bed, only requiring a small tunnel incision [26]. Our results demonstrated a clear difference in the degree of injury and in the time to recovery of tear function between SMILE and FS-LASIK, as assessed by the TBUT. SMILE not only showed a more rapid recovery of tear function, with a return in TBUT to preoperative levels at 6 months compared to 12 months for FS-LASIK, but also showed a significantly lower degree of loss in TBUT compared to FS-LASIK at 3, 6, and 12 months postoperatively. Recent studies have demonstrated that SMILE preserves corneal sensitivity better than LASIK but demonstrated that both procedures eventually result in the recovery of corneal sensitivity to levels seen in healthy controls [9, 21, 25, 30, 43]. This may help to explain the transient nature and the long-term return of TBUT and SEEQ scores to preoperative levels in both groups after 12 months.

Li et al., who examined corneal sensitivity and dry eye following SMILE and FS-LASIK surgery, found that corneal sensitivity was less reduced and thus better in patients that underwent SMILE at all postoperative time intervals compared to those patients that underwent FS-LASIK [41]. The present study found that patients who underwent SMILE not only recovered tear-film function quicker than those who underwent FS-LASIK, but also were less symptomatic in the first 6 months. We also found a moderate correlation between the TBUT and SEEQ scores at 1 month postoperatively in both the SMILE and FS-LASIK groups; however, this correlation did not persist, suggesting that the clinical signs do not always correlate with reported symptoms. This discrepancy between clinical signs and patient symptoms has been previously noted, as Demirok et al. demonstrated that although both SMILE and FS-LASIK resulted in a decrease in corneal sensation up to 3 months postoperatively, there was no change in dry eye symptoms at any point in their patients [25].

Ocular surface changes, including those to the conjunctiva, induced by the two procedures would also differ. Coupled with the effects of hypoesthesia of the cornea, these changes may help to further explain the difference in the development of dry eye disease between SMILE and FS-LASIK patients. Contour changes may impact the distribution of tears over the corneal surface and are likely to pose a greater problem following FS-LASIK than SMILE due to disruption of the epithelium during the formation of the epithelial flap [11, 22]. Damage to and loss of mucin-producing conjunctival goblet cells have been shown to occur following LASIK, resulting in tear-film instability through a reduction of the mucin layer of the tear film [19, 21, 23]. This change may, however, return to baseline after 6 months and may contribute to the transient nature of the postrefractive dry eye disease. An increase in the osmolarity of tears following refractive surgery has also been demonstrated to occur after LASIK [19, 33, 34]. Hyperosmolarity of the tears occurs due to decreased blinking and increased evaporative loss of tears, reduced secretion of tears from the lacrimal gland, and the loss of goblet cells producing the mucin layer of the tear film [19]. This hyperosmolar environment results in the triggering of an inflammatory cascade with an upregulation of inflammatory cytokines, leading to continuing ocular surface irritation, a reduction in TBUT, and the development of dry eye symptoms [33].

FS-LASIK has been proven to be a safe and successful procedure for the surgical correction of refractive error over a number of years [8, 46]. SMILE, although in its clinical infancy, is now proving to also be a safe and successful alternative for the correction of refractive error and may provide a more superior and safer refractive outcome than FS-LASIK [36]. Extensive literature exists demonstrating the safety, efficacy, and complications of FS-LASIK, including a substantial amount of literature investigating the development of ocular surface and dry eye disease following FS-LASIK [8, 10, 11, 17, 19, 22, 23, 47]. Recently, there have been limited studies investigating the development of dry eye following SMILE, as well as studies comparing the two procedures [9, 25, 36, 41]. The majority of the literature, however, has focused on the short-term dry eye outcomes following SMILE and FS-LASIK, looking at the development of dry eye disease up to 3 to 9 months postoperatively. The present study advances on the current literature by investigating and comparing the clinical and subjective dry eye outcomes of patients undergoing SMILE and FS-LASIK over a longer period of follow-up, with measures up to 12 months postoperatively. One other advantage of the present study is that all patients investigated had a moderate to high degree of myopia prior to surgery. This is significant as the large majority of patients undergoing refractive surgery each year are myopic, and the greater the degree of myopia, the greater the amount of stromal ablation or lenticular extraction required [8, 12, 16]. This is a potential area of future research, investigating the development of dry eye disease in relation to the degree of refractive error.

Future advancements can be made on this study to further investigate both the clinical and subjective dry eye outcomes following SMILE and FS-LASIK. The present study was limited in the scope of assessments it conducted, looking at only 2 clinical indicators of dry eye: one objective measure using the TBUT and one subjective evaluation of patient symptoms using the SEEQ. A more comprehensive combination of assessments, as suggested by the Dry Eye Workshop and other studies, would provide a more accurate diagnosis of dry eye disease [5, 24, 37–39]. This would include a measure of tear osmolarity, corneal sensitivity, TBUT, a measure of tear function such as the Schirmer's test, and with advances in technology even the use of confocal microscopy. Also, a more rigorous questionnaire should be utilised, such as the 12-item Ocular Surface Disease Index (OSDI) or the 57-item Impact of Dry Eye on Everyday Life (IDEEL) questionnaires, which have been shown to be more accurate indicators of dry eye disease [2, 47, 48]. The SEEQ had the advantage of being quick and easy to administer, with only 6 items, but has been shown to be outdated and having a low correlation with dry eye signs [47].

The present study demonstrated that SMILE resulted in a lesser degree of dry eye disease and a faster recovery of tear function compared to FS-LASIK in the short-term following surgery in those patients with no preexisting dry eye disease. This was found using both clinical and subjective measures of dry eye and also demonstrated that the long-term outcome was not significantly different between the two procedures after 12 months of follow-up postoperatively. This short-term change, however, can have a great impact on a patients' overall satisfaction with their surgical and visual outcome and may influence their quality of life. Further studies may aim to determine preventative measures that may be taken to help prevent or reduce the development of dry eye disease in patients undergoing refractive surgery and help better monitor and manage those that do.

## Figures and Tables

**Table 1:** Demographic data of the subjects included in this study.

	Mean ± standard deviation		*p* value
	SMILE (*n* = 47)	FS-LASIK (*n* = 43)	
Age (y)	25.21 ± 6.51	24.72 ± 6.53	0.722
Gender (F/M)	30/17	27/16	0.157
Preop SE (D)	−7.46 ± 1.11	−7.44 ± 1.13	0.948
Preop CCT (*μ*m)	546.49 ± 25.52	544.88 ± 24.28	0.761
Preop TBUT (sec)	9.87 ± 1.57	9.56 ± 1.35	0.313

**Table 2:** Lenticule thickness/ablation depth.

	Mean ± standard deviation		*p* value
	SMILE (*n* = 47)	FS-LASIK (*n* = 43)	
Lenticule thickness/ Ablation depth (*μ*m)	138.63 ± 8.56	137.77 ± 13.31	0.711

**Table 3:** TBUT between SMILE and FS-LASIK.

Postop TBUT (sec)	Mean ± standard deviation		*p* value
	SMILE (*n* = 47)	FS-LASIK (*n* = 43)	
1 month	6.28 ± 1.35	6.53 ± 1.24	0.348
3 months	8.21 ± 0.95	7.42 ± 0.96	<0.001
6 months	9.57 ± 0.93	8.19 ± 1.45	<0.001
12 months	9.83 ± 0.99	9.30 ± 0.89	0.009

**Table 4:** SEEQ scores between SMILE and FS-LASIK.

Postop SEEQ	Mean ± standard deviation		*p* value
	SMILE (*n* = 47)	FS-LASIK (*n* = 43)	
1 month	0.77 ± 0.73	1.56 ± 0.63	<0.001
3 months	0.34 ± 0.56	1.21 ± 0.51	<0.001
6 months	0.13 ± 0.34	0.49 ± 0.55	<0.001
12 months	0.04 ± 0.20	0.14 ± 0.14	0.109
